# Data Ownership in the AI-Powered Integrative Health Care Landscape

**DOI:** 10.2196/57754

**Published:** 2024-11-19

**Authors:** Shuimei Liu, L Raymond Guo

**Affiliations:** 1 School of Juris Master China University of Political Science and Law Beijing China; 2 College of Health and Human Sciences Northern Illinois University Dekalb, IL United States

**Keywords:** data ownership, integrative healthcare, artificial intelligence, AI, ownership, data science, governance, consent, privacy, security, access, model, framework, transparency

## Abstract

In the rapidly advancing landscape of artificial intelligence (AI) within integrative health care (IHC), the issue of data ownership has become pivotal. This study explores the intricate dynamics of data ownership in the context of IHC and the AI era, presenting the novel Collaborative Healthcare Data Ownership (CHDO) framework. The analysis delves into the multifaceted nature of data ownership, involving patients, providers, researchers, and AI developers, and addresses challenges such as ambiguous consent, attribution of insights, and international inconsistencies. Examining various ownership models, including privatization and communization postulates, as well as distributed access control, data trusts, and blockchain technology, the study assesses their potential and limitations. The proposed CHDO framework emphasizes shared ownership, defined access and control, and transparent governance, providing a promising avenue for responsible and collaborative AI integration in IHC. This comprehensive analysis offers valuable insights into the complex landscape of data ownership in IHC and the AI era, potentially paving the way for ethical and sustainable advancements in data-driven health care.

## Introduction

Integrative health care (IHC), which emphasizes a holistic approach to patient well-being [1], increasingly incorporates artificial intelligence (AI) to enhance health care delivery. Within this intersection, questions regarding data ownership become pivotal [2]. The wealth of patient data created during the practice of IHC and processed by AI includes medical history, social determinants of health (SDOHs), lifestyle factors, and treatment responses. These underscore the importance of clarifying ownership and control over this sensitive information. Varied legal and ethical perspectives on health data ownership have emerged. These include individuals retaining certain rights and interests in the data, leading to the dilemma of data ownership in health care [3], especially in the age of AI. For instance, in common law countries like the United States, health care providers typically own physical patient records, not patients. While patients have access rights under privacy or freedom of information laws (and health information laws in some regions), these do not equate to ownership. Similarly, government agencies (eg, disease registry administrators) own patient data stored in their databases. In this context, we focus on a detailed exploration of data ownership in the IHC and AI era, discussing its implications and challenges in the United States.

Many countries grapple with data ownership frameworks, particularly in health care. Some, like those with national electronic health records, prioritize centralized accessibility [4,5]. Others, with federalized health care systems, navigate the division of responsibility between national and regional governments [6]. This complexity underscores the need to examine the US context. Within the United States itself, data ownership can also vary significantly. Just like the health care example, some regions might have more centralized data access, while others operate with a more fragmented system. This internal diversity emphasizes the importance of exploring such frameworks across the US landscape.

This study aims to evaluate common data ownership models that apply to the IHC setting, scrutinizing their appropriateness based on the purpose of law and ethics. Through this exploration, we intend to contribute to the ongoing dialogue surrounding the responsible integration of AI in IHC. The study sheds light on the significance of patient-centric data governance, providing insights into the legal and ethical implications and considerations for ensuring responsible and transparent AI implementation within integrative health (IH).

## IH Model

IHC embraces a holistic approach to wellness and centers on the interconnectedness of the mind, body, and spirit, advocating for comprehensive healing that addresses all facets of an individual’s health [1]. IHC often adopts an interdisciplinary modality, fostering collaboration among practitioners from diverse fields to deliver optimal care. This integrative team may include medical doctors, nurses, acupuncturists, chiropractors, and other health care professionals [7]. The IHC model has gained significant momentum in recent years. The United States National Institutes of Health established a dedicated center, the National Center for Complementary and Integrative Health, for IHC research. Additionally, the Veteran’s Health Administration’s Whole Health System [8] exemplifies its implementation within large health care systems. This growing recognition positions IHC as a key component of the learning health system, aiming to continuously improve patient-centered care through data-driven insights [9].

At the core of IHC is integrating conventional and complementary approaches, forming a coordinated health care ecosystem. This approach emphasizes multimodal interventions, combining conventional health care practices (medication, physical rehabilitation, and psychotherapy) with complementary health approaches (such as acupuncture, yoga, massage, lifestyle coaching, and so on) [1]. These tailored interventions address the entire individual rather than focusing solely on a specific organ system. IHC seeks to deliver comprehensive health care that addresses an individual’s well-being by promoting well-coordinated care among diverse providers and institutions.

Incorporating integrative approaches to health and wellness is gaining momentum within health care settings in the United States [10]. Researchers are actively investigating the potential benefits of IHC in diverse contexts, such as pain management for military personnel and veterans, symptom alleviation for patients with cancer and survivors, and programs promoting healthy behaviors [11]. These ongoing investigations seek to shed light on the transformative potential of IHC in enhancing patients’ overall well-being, and patient and provider-generated data often appear through the processes.

An IH practice process typically involves the following steps:

1. The patient consults with an IH practitioner to discuss their health concerns and goals.
2. The IH practitioner assesses the patient’s health and devises a treatment plan that may incorporate a blend of conventional and IH practices.
3. The IH practitioner may recommend the patient to other providers, such as chiropractors, acupuncturists, or nutritionists.
4. The patient collaborates with these additional providers to develop a more comprehensive treatment plan.
5. The health care providers work together to offer the patient the best possible care, focusing on the specific condition and overall well-being and long-term health outcomes improvement.

For instance, a patient experiencing chronic pain might consult an IH practitioner who crafts a treatment plan encompassing physical therapy, acupuncture, and herbal supplements. The practitioner may also recommend a nutritionist to guide the patient in making dietary changes, supporting their healing, and using food as medicine [12] to ease the mental response to the body changes. The IH model is increasingly embraced in health care, reflecting a growing interest in holistic approaches to address health concerns. The interdisciplinary collaboration among IHC practitioners enables patients to receive comprehensive and effective care. However, it is worth noting that the data generated by patients, providers, and other stakeholders throughout this process can be substantial on a large scale, covering a wide range of data fields and categories (Table 1).

In addition to the 3 main categories mentioned earlier, IH clinical practice may also generate data on the following:

1. Patient-reported outcomes (PROs): Include assessments such as pain scales, mood evaluations, and sleep diaries
2. Clinical laboratory tests: Involve procedures like blood work and imaging studies
3. Patient engagement and adherence to treatment plans: Monitoring how actively and consistently patients participate in and follow their treatment plans
4. Cost-effectiveness of IH interventions: Evaluating IH approaches’ economic efficiency and value
5. Adverse events or side effects: Tracking any negative reactions or undesirable effects resulting from IH therapies

IHC represents a paradigm shift in the health care model, moving from a disease-centered approach to a holistic one that prioritizes patient’s well-being by incorporating a wider range of data sources, fostering interdisciplinary collaboration, and potentially leveraging AI for personalized insights. IHC’s strength lies in its interdisciplinary nature, where various providers (doctors, therapists, and nutritionists) collaborate and continuously learn from patient data. This collaborative approach, while fostering innovation and new interventions, creates a complex data landscape. Since IHC collects a wide range of data—medical history, behaviors, SDOHs, and even patient-developed wellness plans (and often uses AI to analyze it), the ownership of these combined datasets and the potential new knowledge derived from them becomes unclear. Furthermore, the very process of AI analysis creates additional complexities. As AI identifies patterns and trends within this rich patient data, new knowledge may be generated. Who owns these “derived data”? Does the ownership lie with the patient who provided the original information or with the platform that developed the AI creating the insights?

These uncertainties around data ownership can discourage patients from fully engaging with IH programs, fearing a loss of control over their personal health information. Clear data ownership policies and legal frameworks are essential to navigate these complexities. The health care stakeholders, including patients, caregivers, providers, and health care systems, need to understand how their data are being used, who has access to it, and for what purposes. Only then can IHC unlock its full potential for holistic and personalized medicine and improved health outcomes while ensuring patient trust and privacy. Next, we will dissect the data ownership issues from the practice perspective.

**Table 1:** Data types generated in the clinical practice.

Category	Examples
Patient information	This type of data can include demographic details, medical history, family history, lifestyle factors, and SDOHs^a^.
Treatment details	This encompasses integrative health therapies provided, including acupuncture, massage therapy, herbal medicine, nutritional counseling, and mindfulness practices. It also includes information on these treatments’ frequency, duration, and intensity.
Patient outcomes	This involves assessing the impact of integrative health interventions on patient health and well-being. Relevant outcome measures include symptom management, quality of life, functional status, and overall satisfaction with care.

^a^SDOH: social determinant of health.

## Data Ownership Issues Rooted in the IHC Practice

Data ownership issues can arise at all stages of health care, including the IHC practice process, from the initial assessment to developing a treatment plan to monitoring the patient's progress.

### Assessment Phase

During the assessment phase, the IHC practitioner may collect data from the patient’s electronic medical records (EMRs), diagnostic tests, and questionnaires. Like other medical fields, data collected from EMRs and tests are often used to plan treatment options. In the IHC settings, PRO data, or real-world data, have more essential roles than traditional models, as PRO data may assist shared decision-making [13] and are associated with the enrollment of IHC approaches [14]. However, from the data ownership perspective, these data may contain sensitive information about the patient’s health condition and lifestyle.

For example, an IHC practitioner may ask patients about their diet, exercise habits, sleep patterns, stress levels, and use of herbs and other supplements that the local policy or law may not regulate. The sensitivity of this information may burden some providers when certain patients have situations that they may not want to share with other stakeholders to avoid potential troubles. For instance, a patient might be hesitant to disclose a history of substance abuse or mental health concerns, fearing discrimination from employers or insurers. This is especially concerning in the context of IHC, where expanded access to data raises the stakes for patients who could face negative consequences for past medical decisions, such as declining to seek treatment or noncompliance. These data are essential for the practitioner to develop an accurate and effective treatment plan for the patient Determining how these data can be used for medical diagnosis, how patients can authorize providers to access them, and how patients can control or participate in data transfer and usage raises significant challenges. However, it is also important to note that these data are sensitive and confidential. The patient has a right to know how their data are being used and to control who has access to it.

### Treatment Plan Development

Once the practitioner has assessed the patient’s health, they will develop a treatment plan. This plan may include a combination of conventional and IH practices, as discussed above. For example, an IHC practitioner may recommend that a patient with chronic pain take a combination of over-the-counter pain relievers, acupuncture, and yoga. The practitioner may also suggest dietary changes and stress management techniques.

The treatment plan may require the patient to share additional data with the practitioner, such as their treatment response or progress. Many of these data may not be categorized or can be innovative exploratory work that no other medical field has touched, which reflects the whole person-based care model. These data can be generated through new interventions, while the treatment plan outcomes formulated between patients and interdisciplinary providers may not have standard end point criteria. Another challenging part here is understanding the different data levels related to a person’s life. Beyond that, much data can be associated with SDOH [15] and other measurements that have yet to be invented or discovered, which could pose future privacy challenges. For instance, the integration of genetic data or continuous environmental monitoring could create more detailed profiles, raising new questions about who has access, how it can be used, and the potential for discrimination [16].

### Monitoring Phase

During the monitoring phase, the practitioner will track the patient’s progress and adjust the treatment plan as needed. This may involve collecting additional data from the patient, such as their symptoms, quality of life, and satisfaction with the treatment.

These data are essential for the practitioner to ensure that the patient receives the best possible care. However, it is also important to note that these data are personal, sensitive, and confidential; like the data generated during the treatment phase, they can be collected by multiple providers. It may also contain health data collected from the patient’s family member and caregiver, including qualitative data that may have the patient’s family member’s personal information. Thus, the patient has a right to know how their data are being used and to control who has access to it.

## AI in the IH Setting

AI technologies have proven to be powerful tools in many health care fields, leading a shift in health care delivery focusing on the patient and their overall well-being [17]. AI holds immense potential to revolutionize the IHC [2] by enhancing patient outcomes, boosting efficiency, and transforming health care delivery [18]. One of AI’s key contributions lies in enabling personalized medicine. By analyzing patient data encompassing medical history, genetics, lifestyle, and environmental factors, AI can assist IHC providers in tailoring treatment plans to individual needs, ensuring optimal care [19-21]. In addition, AI can analyze patient data to identify patterns or markers indicative of underlying health conditions, facilitating earlier and more accurate diagnoses [22]. AI’s capabilities extend to improving treatment outcomes by providing real-time analysis of patient data and presenting relevant treatment options, empowering IHC practitioners to make informed decisions [23]. Furthermore, AI can automate routine tasks, such as scheduling appointments and managing patient records, alleviating the administrative burden on practitioners and allowing them to devote more time to patient care [24,25].

Significant breakthroughs in AI and IHC primarily focus on optimizing therapeutic models, including AI-assisted acupuncture [26], traditional Chinese medicine diagnoses through tongue and lip analysis [19], and traditional Chinese medicine syndrome identifications [27]. In addition, the research explores the use of AI in mindfulness practices [28] and medication adherence [29], leveraging EMR and natural language processing to improve the syndrome pattern diagnosis of lung diseases in integrative medicine [19]. While these clinical trials and applications demonstrate promising progress, other endeavors strive to leverage AI’s advantages in IHC beyond improving existing models, such as patient education and AI-powered symptom analysis. To summarize, the potential applications of AI in IHC can be followed by the 3 phases of IHC—assessment, treatment, and monitoring.

### Assessment Phase

#### Personalized Risk Assessment

AI can analyze vast patient data, including genetic, lifestyle, and environmental factors, to identify individuals at higher risk of developing chronic diseases or adverse health outcomes [30]. This personalized risk assessment can guide preventive health care measures and early interventions.

#### Symptom Analysis and Pattern Recognition

AI-powered tools can analyze patient-reported symptoms, medical history, and clinical data to identify patterns and potential underlying conditions [31]. This can help clinicians make more accurate diagnoses and tailor treatment plans accordingly.

Mental Health Assessment and Screening

AI-based chatbots and virtual assistants can engage in conversations with patients to assess their mental health status and identify potential signs of depression, anxiety, or other mental health concerns. This can facilitate early intervention and support [32].

### Treatment Phase

#### Personalized Treatment Planning

AI can analyze patient data and clinical guidelines to generate personalized treatment plans considering individual factors, including genetic predispositions, past treatments, and coexisting conditions [30,33]. This can optimize treatment efficacy and minimize side effects.

#### Drug Dosage Optimization

AI can analyze patient data and medication profiles to determine the optimal dosage for prescribed medications, reducing the risk of adverse drug reactions and improving treatment outcomes [34,35].

#### Nutritional Guidance and Meal Planning

AI-powered tools can analyze individual dietary needs, preferences, and health goals to provide personalized nutritional guidance and meal planning recommendations, supporting a healthy lifestyle and disease management [36].

### Monitoring Phase

#### Real-Time Remote Monitoring

AI-enabled wearable devices and sensors can continuously collect patient data, such as vital signs, activity levels, and sleep patterns, and transmit it to health care providers for real-time monitoring [37]. This allows for early detection of potential health concerns and timely interventions.

#### Predictive Analytics for Disease Exacerbation

AI can analyze patient data and identify patterns that predict potential disease exacerbations or adverse health events, enabling proactive interventions and preventing complications [24,30].

#### Patient Engagement and Adherence support

AI-powered chatbots and virtual assistants can engage with patients, provide reminders, and offer personalized support to improve medication adherence and lifestyle modifications, enhancing treatment outcomes [38,39].

### Data Generated in AI-Incorporated IHC

With the potential applications of AI in IH clinical practice, the types of data generated can expand beyond traditional patient information and treatment details. Here are some examples of data that can be created with AI integration:

1. Patient-generated health data: AI can analyze data from wearable devices, fitness trackers, and patient-reported symptom trackers to provide insights into patient lifestyle, sleep patterns, and overall health status. These data can be used to personalize treatment plans and monitor patient progress [40,41].
2. Real-time biofeedback data: AI can analyze biofeedback data from devices that measure heart rate variability, skin conductance, and other physiological signals [42]. These data can be used to assess patient stress levels, anxiety, and pain, allowing for real-time adjustments to IH interventions.
3. Genomic and proteomic data: AI can analyze genetic and protein expression data to identify individual variations in drug metabolism, disease susceptibility, and response to IH therapies [43]. This information can tailor treatment plans and predict potential adverse reactions.
4. Predictive analytics: AI can analyze historical data and patient characteristics to predict the likelihood of future health events or treatment outcomes [44]. This information can be used to proactively identify patients at risk and tailor preventive care or treatment plans.
5. AI-driven treatment recommendations: AI can analyze patient data and clinical guidelines to provide personalized treatment recommendations, including the type, dosage, and frequency of IH therapies [30,45]. This can streamline treatment planning and improve patient adherence.
6. AI-powered clinical decision support: AI can provide real-time clinical decision support to health care providers, suggesting appropriate IH therapies based on patient data and evidence-based guidelines. This can enhance clinical decision-making and improve patient care [30,46,47].
7. AI-powered research and clinical trials: AI can facilitate the design, analysis, and interpretation of clinical trials in IH, leading to faster advancements in evidence-based practice [48,49].

## Data Ownership Issues in the IHC or AI Setting

IHC and AI data collection raise complex ownership concerns due to several factors. First, individual contributions to these systems are often intertwined, making it unclear who truly “owns” the resulting data. Second, machine-generated data and AI-derived insights introduce new questions about who holds rights to these intellectual creations. Finally, traditional legal frameworks like copyright and privacy struggle to adapt to the unique dynamics of IHC and AI, leaving ownership ambiguous and potentially sparking disputes. Furthermore, there are several other challenges:

1. Ambiguity around consent and control: Individuals interacting with IHC and AI systems may struggle to understand how their data are collected, used, and shared. Consent mechanisms might be opaque, leaving users unsure if they retain any control over their information [50].
2. Difficulty attributing authorship and creativity: As AI systems increasingly contribute to data generation and analysis, it becomes challenging to determine who deserves credit for the resulting insights [51-53]. Is it the human who provided the initial data, the developer who created the AI, or the AI itself?
3. Balancing individual rights with collective benefits: While data collected through IHC and AI can offer societal benefits like improved health care or personalized services, these advantages can come at the cost of individual privacy and autonomy. Striking a balance between these competing interests remains a significant challenge [50,54].
4. Exploitation and bias risks: Unethical actors might exploit data ownership ambiguities to manipulate or discriminate against individuals [55]. Biased algorithms trained on skewed datasets can further perpetuate such injustices.
5. International complexities: Data ownership laws and regulations vary significantly across jurisdictions, creating challenges for global IHC and AI projects [56]. This can lead to confusion and hinder responsible data governance.

Addressing these complex issues requires ongoing collaboration between technology developers, policy makers, legal experts, and the public. We can ensure equitable data ownership and responsible AI development that benefits all through open dialogue and innovative solutions.

## Who Has the Right to Own the Data?

Data are more critical than ever in the AI and machine learning era. This is especially true in IHC, where AI or machine learning can be used to develop new treatments, improve patient care, and conduct research. However, the ownership of IHC data is a complex issue. There are several stakeholders who may claim ownership of IHC data.

First, patients may argue that they own their data, including data derived from their medical records and diagnostic tests. Traditionally speaking, patients have limited control over the data once the deidentified data are shared with a broader audience. Due to legislation mandating patient privacy, health care providers, institutions, and governing bodies establish policies and practices that determine patients’ ability to access and control their personal health information [57].

Second, IHC providers may argue that they own data derived from their patient interactions, such as data from clinical notes and patient portals. Furthermore, a provider can claim the data ownership if collected from a new intervention or clinical trial. The interaction between patients and providers is also meaningful, as patients can refuse to share the data in any research capacity. While data privacy laws allow patients to control who has their data and how they are used, limited knowledge about existing data holdings creates an informational asymmetry, hindering their ability to fully exercise these rights [58]. Thus, health care providers may proactively opt patients out of broad research programs at the outset to address limited patient control over data reuse [59]. While opting out can prevent future data collection, it does not necessarily erase existing data held by researchers, government agencies, or private entities. This creates a situation where patients may struggle to exercise their data privacy rights due to limited knowledge of which entities hold their data.

Third, researchers may argue that they own data from their research, including data derived from IHC patient data and interventions. Many clinical providers are involved in linear research activities, which often follow the health care stream from identifying diseases to developing interventions. When considering the application of AI in clinical practice, the researchers may claim ownership of the developed algorithm (through a patent); however, in some circumstances, they may claim ownership of the data being used in the research trials and projects.

Fourth, AI and machine learning developers may argue that they own the data to train AI or machine learning algorithms, including IHC patient data, as some deidentified personal data can be purchased or licensed for research depending on its availability and approval processes. However, access to other sensitive health data, particularly from IHC settings, is often more restricted. These datasets may require specific approval from research ethics committees before access is granted [60,61].

Finally, technology companies may argue that they own data collected through their wearable devices and other health-tracking apps, including IHC patient data. Multiple companies can claim over the same data collected at the same time.

## Data Ownership Models Related to IHC

Data ownership models in health care are a complex and evolving topic. There are a variety of different models, each with its advantages and disadvantages. Some of the most common data ownership models in health care are described in this section.

### Privatization Postulate

Originating from John Locke’s natural rights theory, the privatization postulate in health care data ownership asserts that data are a valuable private asset owned and controlled by individuals or organizations [62,63]. Within the context of IHC and AI collaboration, this model raises concerns about private entities’ potential monetization of IHC data. This practice could hinder interdisciplinary collaboration, as apprehensions regarding data protection might limit information sharing among health care providers. Furthermore, developing AI under the privatization postulate may lead to proprietary algorithms, restricting their accessibility and hindering the collective advancement of IHC treatments. The focus on individual or organizational ownership may create barriers to the seamless sharing of insights and innovations, impeding the collaborative potential of IHC in the era of AI.

Despite these challenges, the privatization postulate does offer advantages. It recognizes the economic value of health care data, potentially incentivizing individuals, and organizations to invest in data collection and analysis. This could lead to advancements in personalized health care solutions and tailored treatment plans. However, the drawbacks lie in the potential negative impact on collaboration, data accessibility, and collective progress in the IHC landscape. Striking a balance between recognizing the value of data as an asset and fostering collaborative efforts is crucial for successfully integrating AI in health care under this ownership model.

### Communization Postulate

Unlike the privatization postulate, the communization postulate views data as a public good to be shared openly, and data can be used simultaneously and legally [63-65]. In the context of IHC and AI, this model emphasizes collaboration and coordination among interdisciplinary health care providers, researchers, and patients. The concept of shared data platforms and open-source AI aligns to improve resource use and, consequently, patient outcomes. Challenges may arise while this model envisions a more inclusive and collective approach to health care data. Concerns about responsible and ethical AI use and the equitable sharing of benefits necessitate careful consideration. Achieving a balance between open collaboration and addressing ethical concerns becomes imperative to realize the positive outcomes envisioned fully under the communization postulate.

The advantages of the communization postulate lie in its potential to break down data silos, promoting seamless data sharing and accessibility among health care providers. This collaborative environment can foster innovation, leading to more effective IHC treatments. However, the model also raises ethical considerations, such as ensuring data are used responsibly and equitably [63]. Striking this balance is crucial for successfully implementing the communization postulate in IHC, ensuring that the benefits of shared data extend to all stakeholders while upholding ethical standards.

### Intellectual Property

Ownership of health data can be both tangible and intangible property [66]. Regarding tangible property rights, the answer is sometimes for sure. For example, it is likely to be said that medical providers, rather than patients, typically own physical medical records in the United States [3]. Meanwhile, health data are intangible information. Relevant stakeholders can own health information based on different types of laws in the field of intellectual property, including patent law, copyright law and copyright in databases, trademark law, and trade secrets. However, such ownership protection must meet various criteria, leading to clarity and incomplete or partial ownership protection of health data [66]. For example, health data should be patent eligible in order to enjoy patent protection [67]. Also, trade secrets or relevant confidential information laws apply to limited types of health data and several questions are still open concerning ownership of health data [68]. Furthermore, conferring ownership rights through intellectual property law is even more complicated in the AI background, such as AI’s capacity to claim intellectual property rights, determining contributions between humans and AI, and so forth. Therefore, answering ownership questions concerning AI-generated health data in the context of intellectual property law is highly complex and uncertain.

Next, we will discuss the current data ownership models in health care data that are related to IHC. Understanding the advantages and disadvantages of these models can help to address the rising issues and conflicts in the data ownership of IHC in the AI era.

### Distributed Access Control Model

The distributed access control (DAC) [69] model presents a decentralized approach to data ownership, providing individual health care providers or organizations more control over their data, especially in the context of IHC and AI. This model addresses critical concerns related to privacy and security in health care data. By allowing entities to control access to their data through mechanisms such as role-based access control or attribute-based access control, the DAC model aims to safeguard sensitive patient information. However, the emphasis on individual control may lead to challenges in data sharing between providers, resulting in fragmented care and potentially hindering medical research progress within the interdisciplinary landscape of IHC.

While the DAC model helps mitigate privacy and security concerns, it introduces complexities related to data silos and barriers to efficient information exchange. The fragmented nature of data ownership under DAC can hinder collaborative efforts in IHC, limiting the comprehensive understanding of patient health and potentially compromising the effectiveness of treatments. In addition, difficulties in research access may arise, as researchers need permission from each provider or organization that owns the data. Balancing individual control with the need for seamless collaboration and research access becomes essential in implementing the DAC model effectively within IHC, particularly in the era of AI.

### Data Trusts

Data trusts, as legal entities holding data for multiple stakeholders, offer an alternative to the DAC and communization models in the landscape of IHC and AI [26]. In this context, data trusts provide increased control over data for stakeholders, promoting responsible and ethical use. Establishing a neutral and trusted third party to manage data helps address data ownership, privacy, and security concerns. However, challenges persist, particularly regarding the complexity and cost of setting up and maintaining data trusts in IHC. The intricacies involved in creating and maintaining these legal entities may not be feasible for all IHC providers, limiting the universal adoption of this model.

Despite the potential advantages, such as improved data sharing and collaboration, data trusts may face difficulties aligning the interests of the trust and stakeholders. Conflicts over data ownership and use could arise, highlighting the importance of establishing clear guidelines and frameworks for the functioning of data trusts in the realm of IHC and AI. In addition, holding data trusts accountable for their actions may prove challenging due to their complex and opaque nature. Striking a balance between the benefits and challenges of data trusts becomes crucial for their effective integration into the IHC landscape, ensuring that they contribute positively to data management and use in the era of AI.

### Blockchain Technology

Blockchain technology emerges as a promising solution for secure and transparent data ownership records, particularly in the context of IHC and AI. In health care, blockchain could enhance transparency, accountability, and data sharing, reducing the risk of breaches and other security incidents [70]. However, concerns persist about the scalability and reliability of blockchain technology, mainly when applied to manage large amounts of health care data within the interdisciplinary collaboration inherent in IHC. The relatively new nature of blockchain introduces uncertainties about its widespread implementation and integration into existing health care systems.

The advantages of blockchain in IHC include its potential to create an immutable and tamper-proof ledger, ensuring the integrity of health care data. This can be particularly beneficial in maintaining accurate patient records and supporting collaborative efforts among health care providers. However, the complexity and cost of implementing blockchain technology may pose challenges, especially for smaller IHC providers with limited resources. The lack of a clear regulatory framework adds to the complexity, introducing uncertainties about data ownership and usage within the IHC landscape. Furthermore, data privacy laws, both common law and civil, are often incompatible with public blockchains because anyone can see the information stored on them. This transparency can be a major issue for sensitive data. To help developers navigate this challenge, the National Institute of Standards and Technology has created a flowchart to identify suitable blockchain use cases. Striking a balance between leveraging the benefits of blockchain and addressing the challenges is essential for its successful integration into the evolving landscape of IHC and AI.

## Rethinking the Data Ownership Framework of IHC Practice in the AI Era

While IH offers a promising shift toward holistic patient well-being through collaboration and AI-powered insights, the complex data landscape it creates necessitates a robust data-sharing model. The current lack of clarity around ownership of combined datasets and AI-derived knowledge discourages patient participation and hinders progress. Addressing these concerns involves more than just technical solutions; it requires a data ownership model to safeguard data ownership rights.

Further clarification and specification of data ownership from a legal perspective is essential to consider the recommendations and legal action. Property is not the object itself but rather the ability to assert control through aggregated legal interests as McGuire et al [3] pointed out [71]. Ownership encompasses legal rights, including but not limited to possession, access, and control. Despite opposing views on establishing property rights in data for reasons such as public good, lack of market failure, fundamental rights, and transaction costs [64], it is crucial to promptly assign health data ownership. This step is necessary to actively incentivize the high-quality, efficient generation, dissemination, and use of medical data, thus energizing AI development in IH settings.

Simultaneously, the bestowed ownership of health data should be limited to strike a balance among various stakeholders’ interests and appropriately reduce transaction costs. Limiting ownership for different entities aligns with the law’s purpose of promoting societal progress and maintaining balance. As discussed earlier, patients, IHC providers, researchers, and AI or machine learning developers all contribute to IHC practice data in the AI era. Co-ownership among these interested parties is essential, but granting full ownership rights to each stakeholder could significantly increase transactional costs, potentially hindering the application of health data in clinical settings, research, and AI fields. Due to the complexity involving numerous rights and interest holders, providing recommendations is challenging.

One suggested framework is to grant patients ownership only over their personal health data. Since health data without personal information have less connection to patients and dealing with numerous patients would dramatically increase transactional costs, restricting ownership to personal health data is a prudent choice. Furthermore, specific legal rules for ownership rights concerning patients’ personal data can be explored with reference to Articles 5-22 of the General Data Protection Regulation (GDPR) [72]. Under GDPR, patients can request access to their own data and have some level of control (eg, delete the data or ask not to be shared with third parties). However, they do not have data ownership, which is similar to other privacy frameworks. Another crucial aspect is to establish limitations or exceptions regarding health data ownership for stakeholders like patients, IHC providers, researchers, and AI or machine learning developers. These limitations and exceptions help balance the interests among stakeholders, drawing inspiration from various fair use models in intellectual property law.

Defining ownership rights and limitations for stakeholders in the context of IHC in the AI age is challenging, especially with the emergence of AI. Identifying and allocating data ownership rights, such as determining ownership based on proportion or primary contributors, present ongoing challenges. Consideration must be given to patients’ rights and privacy, physicians’ efforts, AI practitioners’ involvement and time commitment, and public interests. Only through this comprehensive approach can medical development and the balance of various interests be promoted, aligning with the purpose and spirit of legal regulations. While this is just 1 aspect of many possible recommendations, it is crucial as legal clarity on these issues will directly impact the establishment and implementation of other suggestions to address data ownership in IHC practice in the AI era.

In summary, we propose the Collaborative Healthcare Data Ownership (CHDO) framework. The CHDO emphasizes the collective power of data when stakeholders work together. It acknowledges that various parties contribute valuable insights to health care data, from patients to providers, researchers, and AI developers. The CHDO framework addresses this by proposing three key features:

1. Shared ownership: The CHDO framework goes beyond traditional ownership models, where one entity holds exclusive rights. Instead, it advocates for co-ownership, granting stakeholders specific rights and responsibilities over the data based on their contributions. This fosters trust and incentivizes collaboration, unlocking the full potential of data for research, development, and personalized care.
2. Defined access and control: The CHDO framework advocates the establishment of clear guidelines for accessing and using data. Patients retain control over their personal health information, while other stakeholders can access anonymized or aggregated data for approved purposes. This balance ensures individual privacy while enabling collective advancements in health care.
3. Fair and transparent governance: The CHDO framework recognizes the need for robust governance structures. Transparent policies and procedures ensure equitable access, prevent misuse, and address potential conflicts. This fosters trust and accountability among all stakeholders, creating a sustainable environment for data-driven health care progress.

Based on the analysis of various data ownership models in the context of IHC and AI presented in the previous sections, the CDHO co-ownership model offers several advantages over other frameworks. It addresses the concerns raised by the privatization postulate regarding the impact on public interest by granting patients ownership over their personal health data. In addition, it mitigates the ethical issues that may arise from the communization postulate and the stand-alone DAC model, such as privacy and security concerns. Furthermore, the CDHO model avoids limited ownership protections based on patent law, trade secrets or relevant confidential information laws, copyright law, and trademark law in the context of IHC and AI. It is important to note that the other 2 models, data trusts and blockchain technology, are primarily concerned with the management and storage of health care data and can incur significant costs. In these models, conflicts between stakeholders can persist in the absence of clear ownership rights, and there is no clear guidance for resolving them. Whether trusts or blockchains are used, a prerequisite for their establishment is the clear identification of the party with ownership rights to establish the trust or blockchain. The CHDO model effectively addresses this issue by clearly defining and balancing the interests of all parties, ensuring individual privacy and security, and promoting the realization of public interest.

These advantages are particularly significant in the context of IHC and AI. IHC’s collaborative nature and focus on patient-centered care necessitate a data ownership model that fosters trust and incentivizes collaboration (shared ownership). Furthermore, the need to balance individual privacy with the potential of data for research and development aligns well with the CHDO framework’s defined access and control mechanisms. Finally, the CHDO framework’s emphasis on fair and transparent governance is crucial for navigating the complex ethical considerations surrounding AI use in health care. By implementing these principles, the CHDO framework unlocks a new era of collaboration, empowering stakeholders and fostering a healthier future for all, ultimately propelling the health care industry toward a data-driven and AI-integrated future that prioritizes both individual rights and collective progress.

## Conclusion

While a universal solution may be elusive, navigating data ownership challenges in AI-powered IHC requires tailoring approaches to specific stakeholder needs and regulations. By ensuring that data use benefits individual patients, adheres to legal frameworks, and contributes to societal well-being, this collaboration can unlock the full potential of AI in IHC while mitigating legal risks. In essence, addressing data ownership in IHC can pave the way for a more streamlined, effective, and ethical integration of AI in health care, ultimately amplifying its benefits for the whole society.

## Abbreviations

AI: artificial intelligence
CHDO: Collaborative Healthcare Data Ownership
DAC: distributed access control
EMR: electronic medical record
GDPR: General Data Protection Regulation
IH: integrative health
IHC: integrative health care
PRO: patient-reported outcomes
SDOH: social determinant of health
